# A Review on Revolutionary Natural Biopolymer-Based Aerogels for Antibacterial Delivery

**DOI:** 10.3390/antibiotics9100648

**Published:** 2020-09-28

**Authors:** Esam Bashir Yahya, Fauziah Jummaat, A. A. Amirul, A. S. Adnan, N. G. Olaiya, C. K. Abdullah, Samsul Rizal, M. K. Mohamad Haafiz, H. P. S. Abdul Khalil

**Affiliations:** 1 School of Industrial Technology, Universiti Sains Malaysia, Penang 11800, Malaysia; essam912013@gmail.com (E.B.Y.); ngolaiya@futa.edu.ng (N.G.O.); ck_abdullah@usm.my (C.K.A.); mhaafiz@usm.my (M.K.M.H.); 2 Management Science University Medical Centre, University Drive, Off Persiaran Olahraga, Section 13, Shah Alam, Selangor 40100, Malaysia; drazreenadnan@gmail.com; 3 School of Biological Sciences, Universiti Sains Malaysia, Penang 11800, Malaysia; 4 Department of Mechanical Engineering, Universitas Syiah Kuala, Banda Aceh 23111, Indonesia; samsul_r@yahoo.com

**Keywords:** nanocellulose, aerogel, biopolymer, antibacterial, drug delivery, wound healing

## Abstract

A biopolymer-based aerogel has been developed to become one of the most potentially utilized materials in different biomedical applications. The biopolymer-based aerogel has unique physical, chemical, and mechanical properties and these properties are used in tissue engineering, biosensing, diagnostic, medical implant and drug delivery applications. Biocompatible and non-toxic biopolymers such as chitosan, cellulose and alginates have been used to deliver antibiotics, plants extract, essential oils and metallic nanoparticles. Antibacterial aerogels have been used in superficial and chronic wound healing as dressing sheets. This review critically analyses the utilization of biopolymer-based aerogels in antibacterial delivery. The analysis shows the relationship between their properties and their applications in the wound healing process. Furthermore, highlights of the potentials, challenges and proposition of the application of biopolymer-based aerogels is explored.

## 1. Introduction

The aerogel is a novel material formed by replacing the liquid of a gel with gas without changing its structure. Aerogels were first prepared with a supercritical drying technique using alcohol as a liquid [1]. A few years later, this material was further experimented and utilized in different applications. Abdul Khalil et al. [2] describe an aerogel as a material which contains more than 99% air and can be prepared from various precursor material. Often, aerogel is manufactured in multi-shape structures to serve different needs. The first aerogel was produced from silica gel as a precursor material [3]. Aerogel has since been prepared from organic, inorganic, and even composites of different materials. At present, biopolymers have attracted a lot of research work for aerogel preparation with a focus in biomedical applications. Biomedical application of aerogel includes wound healing, tissue engineering scaffolds and drug delivery [4]. Biopolymers have properties desirable for potential materials used in biomedical applications. These properties include biocompatibility, non-toxic, hemostasis and antibacterial properties. The antimicrobial properties of some biopolymers make them desirable materials in wound healing applications [5]. Much research has been conducted on the preparation of biopolymer-based aerogels and its characterization.

**Figure 1:**
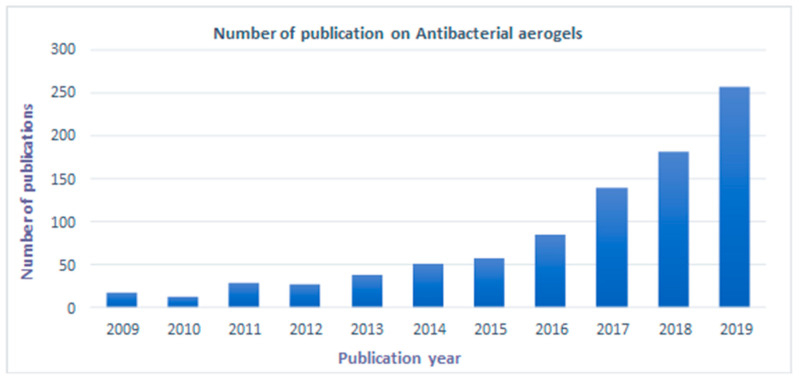
Number of scientific publications in the last ten years, contributing to the subject “biopolymers aerogels” by year (Search conducted through Science Direct on 9 September 2020 (From 2009 to 2019)).

Furthermore, in vitro and in vivo analysis have been conducted to evaluate its use in different applications, including antibacterial delivery. Even with the considerable developments in aerogel preparation, its commercialization is still relatively slow. The number of companies producing aerogels with desired properties for different applications is low. Many authors have proposed with a conviction that a lot of newer aerogels are going to be prepared in the next few years. The interest in biopolymer-based aerogels has grown, as indicated by the number of publications each year as Figure 1 presents.

There are already several excellent review articles and books about the history, properties, medical and non-medical applications of aerogels [6,7,8,9]. Also, the chemistry, physics, synthesis, and their different applications have been extensively discussed [2,10,11]. However, not much has been done explicitly regarding the utilization of biopolymer-based aerogels for antibacterial delivery. This review selectively examined biopolymer-based aerogels used in antibacterial delivery. Biopolymer-based aerogels such as chitosan-based, cellulose-based and alginate-based aerogels were discussed extensively. This review includes an overview of their properties and the most recent works utilizing these aerogels in antibacterial delivery applications. Additionally, the study further discusses the role of biopolymer-based aerogels in superficial and deep wound healing and to stop hemorrhaging. The study also highlighted some of the challenges and propositions of these biopolymer-based aerogels in antibacterial delivery applications.

## 2. Biopolymer-Based Aerogels

Biopolymers defined as naturally occurring materials consist of repetitive monomeric units that covalently bonded to form larger molecules, such as cellulose, collagen, and alginates, etc. In the past few years, biopolymers have been extensively used in aerogel preparation for different biomedical applications due to their unique properties [7]. Aerogel was defined as a solid, ultra-lightweight and lucid open porous network, obtained from gel following the removal of the pore liquid without any significant modification in the network structure [12]. Traditional materials for aerogel preparation like silica, carbon, metals, and synthetic polymers present some drawbacks that limit their use in biomedical applications. These drawbacks include immunological rejection by the body and their high cytotoxicity, which may cause undesirable immunogenic response [13]. The studies on biopolymers are motivated by the search for sustainable precursors instead of using traditional oil-based or synthetic materials [10]. Furthermore, the biopolymers are desired in medical and biomedical applications due to their biocompatibility and non-cytotoxicity.

The first aerogel fabrication approach was used by Kistler, the father of aerogels [14]. He used sodium silicate as precursor material and heated it in an autoclave above the critical temperature with the presence of pressure. This approach later became widely known as supercritical drying [15]. A few years later, a sol-gel approach was designed by Teichner and Nicoloan, where they developed Kistler’s approach by using tetramethyl orthosilicate (TMOS), which was easier to remove than the water glass that Kistler used during supercritical drying [2]. Other methods have been used to fabricate biopolymer-based aerogels including freeze-drying [16], gas foaming [17], electrospinning [18], and thermal-induced phase separation [19], which all have been presented and discussed by Abdul Khalil et al. [6]. Recently, a new generation of computer aid fabrication approaches have been developed, which are called rapid prototyping [20]. Various computer-aided methods have been fabricated to design the desired shape of 3D aerogels using biopolymers as bio-injected ink. Stereolithography [21], selective laser sintering [22], fused deposition modelling [23], and 3D printing [24,25] are examples of the most used fabrication techniques of biopolymer-based aerogels. Chitosan, cellulose and alginate have been used in various medical applications due to their unique biological characteristics such as hemostatic properties, antibacterial and mucoadhesive properties which are useful for wound healing and stopping hemorrhaging. Table 1 presents a summary for the biological wound healing properties and molecular structures of chitosan, cellulose and alginates. These biopolymers have been used in tissue engineering scaffolds, biosensing, medical implants, and drug delivery [2]. The chemistry of aerogels is very flexible and has been reported to be altered by many factors such as the precursor materials [26], their ratio [27], preparation approaches [1] and so on. The pore size and the surface area of aerogels can also be tailored [28]. The physical, chemical and mechanical properties of aerogels mainly depend on the precursor material or group of materials that form this network.

### 2.1. Chitosan-Based Aerogel

**Figure 2:**
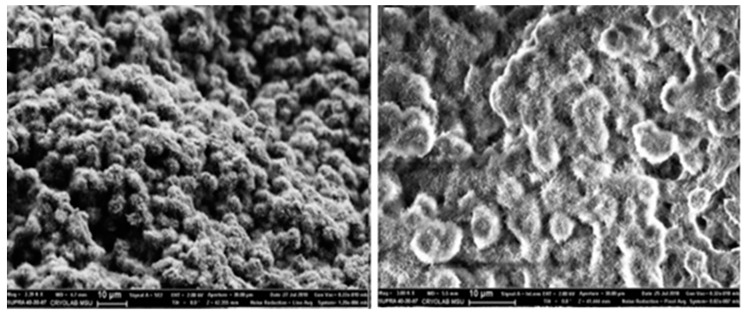
Scanning Electron Microscope images for the chitosan-based aerogel. Adapted from Rubina et al. [39].

Chitosan is one of the most abundant biopolymers on earth and has been extracted from multiple sources such as the shells of crustaceans [32], cell walls of fungi [33], and exoskeleton of arthropods [34] etc. The preparation of aerogels from polymeric nanoparticles such as chitosan is attracting increasing interest among scientists. The unique properties of resulted aerogels, such as high surface area, mechanical strength, a high degree of polymerization, high purity and high crystallinity make them promising materials for various desired applications [35,36]. Chitosan has been reported to be insoluble in water and forms an aqueous alkaline solution. The convenient mechanism resulting from pH-dependent solubility of this polymer allow its processing under mild conditions and the ability to form various shapes and sizes of hydrogel or aerogel [37]. Yi et al. [38] fabricated highly porous and anisotropic chitosan aerogels through directional freezing technology, which can sustain an extremely high compressive strain. The authors reported that the chitosan aerogel was able to completely revert to its original height with the release of imposed weight. Rinki et al. [37] fabricated a chitosan aerogel using supercritical carbon dioxide technique which exhibited a polymorphic mesoporous structure. The authors reported that the use of supercritical CO_2_ technique enhances the porosity of aerogel. The structure of aerogel changed from nonporous and smooth structure to a porous and leaf-like structure. The surface area of the aerogel was in the mesoporous range. In another study, Rubina et al. [39] prepared chitosan aerogel with a noticeably larger globule-like structure, as presented in Figure 2. In a different study, a comparison of the mechanical properties of a chitosan-based aerogel was studied by Gómez et al. [40]. In their work, they compared the mechanical strength of the aerogel after loading it with different materials. The mechanical properties of the aerogel were affected upon loading the antibiotic (erythromycin), the aerogel showed crystalline deposits and a decrease in the hardness and Young’s modulus, unlike elephant garlic extract loaded aerogel, which showed increase on the hardness and Young’s modulus, as well as showing texturization of the micro sheets and microfibers [40]. This reveals that any addition to the aerogel may affect the morphology, mechanical strength, hardness and porosity.

### 2.2. Cellulose-Based Aerogels

**Figure 3:**
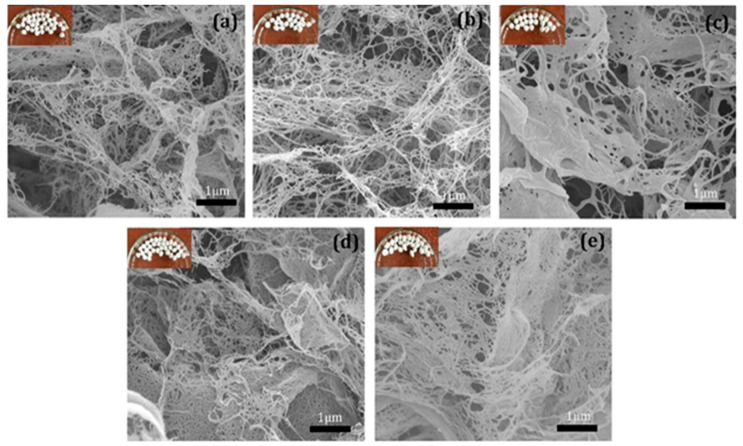
Scanning Electron Microscope images of (**a**) mix 1:3 (cellulose nanocrystal/cellulose nanofiber) aerogel, (**b**) mix 1:1 aerogel, (**c**) mix 3:1 aerogel, (**d**) cellulose nanocrystal aerogel, (**e**) cellulose nano fiber aerogel. Adapted from Zhang et al. [47].

Cellulose is the most abundant biopolymer on earth and has been extracted from a variety of renewable and sustainable sources [41,42,43]. It is found in 9–25% of plant primary cell walls and 40–80% of their secondary cell walls. Cellulose also isolated from bacteria and some animals [44]. Nanocellulose refers to nano-structured cellulose materials, which either cellulose nanocrystal (CNC) or cellulose nanofibre (CNF) [45]. Both forms of cellulose have unique properties such as high surface area, high strength, and tunable surface chemistry. These properties are responsible for controlled interactions with other polymers, other nanoparticles, biological materials or small molecules; thus, it is the most utilized biopolymer in different applications [1]. The properties of cellulose-based aerogel differ based on the materials used; CNF-based aerogel display more rice-shape in morphology compared to the spherical CNC aerogel. The shape of nanocellulose materials affects the appearance of aerogel [46]. Zhang et al. [47] compared the performance of CNC and CNF aerogels and reported that mixing the two materials showed better performance than the pure aerogel resulted from each of them. However, all the aerogels that were prepared had a 3D network structure and rich in pores. Figure 3 presents the obtained SEM images of the aerogels that were prepared by Zhang et al. [47]. The morphology result showed that CNC based aerogel is spherical while the CNF based aerogel showed rice-shape, which resulted from the longer filament of CNF compared to CNC. The size, structure and morphology of aerogel pores can be adjusted by changing the precursor material, its concentration or mixing it with other particles. The mechanical properties of aerogel have been reported to be affected by two main factors: precursor material and preparation method [48]. Yang et al. [49] reported that cellulose enhanced the mechanical properties of the aerogel, and the ratio of alginate cellulose had a significant effect on the mechanical properties. In a different study, Zheng et al. [50] evaluated the mechanical properties of cellulose aerogels by using CNC as reinforcement and concluded that the compressive modulus was significantly improved (6 times higher) compared to the pure cellulose aerogel. Qin et al. [51] prepared cellulose-based aerogel with a specific surface area and a small amount of mesopores. The authors evaluated the effect of loading Resveratrol drug into the aerogel and concluded that smaller amounts of pores in the aerogel appeared richer. Revealing that the appearance of new pores and the changing of pores diameter resulted after the addition of drug [51].

### 2.3. Alginate Based Aerogels

**Figure 4:**
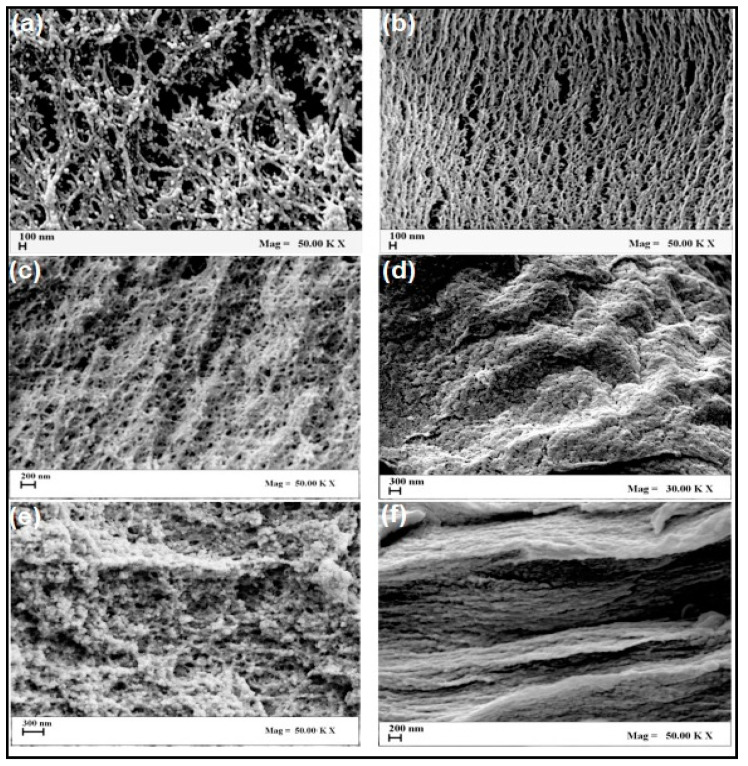
Internal section Scanning Electron Microscope images of: (**a**) 5% *w/w* Ca-Alginate aerogel, (**b**) 5% *w/w* Cu-Alginate aerogel, (**c**) 10% *w/w* Ca-Alginate aerogel, (**d**) 10% *w/w* Cu-Alginate aerogel, (**e**) 15% *w/w* Ca-Alginate aerogel, (**f**) 15% *w/w* Cu-Alginate aerogel. Adapted from Baldino et al. [54].

Alginate is a natural polymer mainly obtained from brown algae and seaweeds. It consists of linear copolymers of β-(1–4) linked d-mannuronic acid and β-(1–4)-linked l-guluronic acid units [52]. Sodium alginate is widely used as a thickening agent in the food industry and antidote for heavy metal ions in medicine [53]. This anionic polysaccharide was able to cross-link with any divalent cation such as Ca^2+^, Zn^2+^ and others. Alginate has been widely used in aerogel fabrication for many medical [54] and non-medical applications [55]. Alginate-based aerogels are reported to possess bulk densities, high surface areas and super absorbent behavior [56]. They have the advantage of being easily processed from educts only by exchange of water, alcohol and carbon dioxide alone without the need for other solvents, which is considered a safe alternative in terms of medical applications and wound dressing [57]. Robitzer et al. [58] studied the organization at the nano-scale level of alginate-based aerogels, and it was concluded that morphology of the aerogel depends on the morphology of pre-existing objects within the gel, and any adjustment of the parent gel will affect the structure of the aerogel. The concentration of the polymeric solution is the main inference on the properties of aerogels. Baldino et al. [54] fabricated cylindrical shape alginate-based aerogels using supercritical drying technique. They studied the effect of polymer concentration on the physical and mechanical properties of aerogel. The authors reported that the morphology of aerogels changed from nano-fibrous to nano-porous when the alginate concentration increased as presented in Figure 4. The results of specific area values indicated that the lower alginate concentration, the higher specific area [54]. Calcium alginate porous aerogels have been prepared in another study following the same technique of supercritical CO_2_ drying. It was observed that the aerogel from calcium alginate formed different pore sizes termed as micropores, macropores and surface area [59]. The pore size of alginate-based aerogels was reported to be of nano-scale, in a recent study by Franco et al. [60]. The characterization of calcium alginate aerogel showed that the aerogel had nano-porous structure using supercritical CO2 drying. Alginate/pectin aerogel microspheres were fabricated by Chen et al. [61] and revealed that higher alginate concentration presented higher stiffness and mechanical strength, while higher pectin concentration presented higher porosity and pore size.

### 2.4. Other Biopolymer-Based Aerogels

Aerogel have been also fabricated from other biopolymers such as pectin [62], starch [63], carrageenan [64], agar [65], and xanthan [10,66]. Nešić et al. fabricated environmentally friendly pectin-based aerogel using TiO_2_ as an antibacterial agent. The authors compared the mechanical and thermal properties of pure pectin and pectin/TiO_2_ aerogels and revealed that the mechanical and thermal performance improved after the addition of metal oxide [62]. Starch-based aerogels have been prepared from different sources such as corn, pea, tapioca and potato [67]. Baudron et al. [68] recently prepared starch-based aerogel with particles size varied from 25 and 270 μm and Brunauer–Emmett–Teller (BET) surface area of 278 m^2^/g. Agar has been extracted from red seaweeds, which mainly composed by two polysaccharides namely; agarose and agaropectin [69]. Agar showed good compatibility with other biopolymers and it have been used to enhance the mechanical properties of starch based aerogels [69]. Carrageenan is another naturally occurring polysaccharide was used in aerogel fabrication, Alnaief et al. [26] used micro-spherical carrageenan particles to fabricate biodegradable aerogel with a surface area ranged between 33 and 174 m^2^/g, and an average pore 12.3 nm.

### 2.5. Biomedical Applications of Biopolymer-Based Aerogels

Biopolymer-based aerogels have been extensively prepared, characterized and experimented for numerous biomedical applications due to their biocompatibility, lesser or non-cytotoxicity [70] and immunological response [71]. Biopolymer has been utilized in biosensing, controlled drug release, tissue engineering scaffolds, skin and tissues repairs, etc. Aerogels have been used to overcome the issues associated with 1D and 2D materials in developing biosensors. Edwards et al. [72] used peptide-nano cellulose aerogels for biosensing. They fabricated an aerogel with high porosity to detect protease enzyme activity. The results of mass spectral analysis and the physical properties of prepared aerogel showed promising potential for wound dressing applications. A biosensor is an analytical device or material that can detect low concentrations of a desirable parameter [2]. Cellulose nanocrystals aerogel has been fabricated to be able to detect human neutrophil elastase for healing chronic wounds [73]. Biosensors, combined with certain biological components, form a physicochemical detector, which could be enzymatic or non-enzymatic based. Li et al. [74] successfully used chitosan-carbon nanotubes hybrid aerogel for non-enzymatic sensing. Peptide/cellulosic aerogel has been used as a micro-titer enzyme assay to determine the response, sensitivity, and kinetic behavior of the biosensor [75].

Tissue engineering is another application of aerogel. Aerogel has been used for regeneration of various types of tissues such as skin, bones, cartilages and even blood vessels. Collagen-alginates aerogel has been evaluated for the regeneration of different tissues [76]; using bio-based materials has the advantage of being cytocompatible and non-toxic. Cellulose-based aerogel has been suggested to solve the issue of the potential complications associated with autografts. It has been widely used in tissue engineering scaffolds preparation. Osorio et al. [77] modified cellulose nanocrystal aerogels and used them as viable bone tissue scaffolds. Their findings demonstrated that cellulose-based aerogels are flexible, porous and facilitate bone growth after they are implanted in bone defects. Alginate/lignin nanocomposite aerogel has been evaluated for tissue scaffold. It possesses good cell adhesion properties and did not compromise cell viability [78]. Using natural materials such as cellulose, alginates and chitosan in tissue scaffolds did not show any toxicity and, in most of the cases, enhanced the growth of cells. Kumari and Dutta [79] fabricated genipin-chitosan hybrid aerogel and evaluated its cytotoxicity. The porous structure of aerogel supported cell adhesion and proliferation. It was reported to be far less toxic than glutaraldehyde and safe for tissue engineering application.

Controlled drug release at present has been at the forefront of research. Biopolymer-based aerogel has been proposed for use in controlled drug release. Rinki et al. [37] evaluated chitosan aerogel for potential drug delivery. The report of the study showed that the aerogel was biocompatible, non-cytotoxic, with intense antibacterial activity, making it suitable for drug delivery applications. Zhao et al. [80] used a combination of poly-ethylenimine grafted in cellulose nanofibril aerogels for drug delivery. It was reported that the material is promising and proposed as a new generation of controlled drug delivery carriers. The unique pH and temperature-responsiveness together with their excellent physical, chemical, mechanical, biodegradability, biocompatibility and less cytotoxicity of biopolymers make them better than synthetic polymers. Alginate aerogel has also been reported to be suitable in mucosal drug delivery, mucoadhesive and non-cytotoxic [81]. The presence of multi-functional groups on the surface of biopolymers and the advantage of the extremely high surface area of aerogel as material make them the right and desirable choice for drug delivery applications. 

## 3. Biopolymer-Based Aerogels for Antibacterial Delivery

Antibacterial delivery is one of the biomedical applications that has attracted a lot of research in the past few years. A significant number of publications have been published, and more research is still being conducted in this area. Drugs or natural antibacterial materials loading in the aerogel can be achieved either by adding them in the sol-gel process or in the post-processing of synthetic aerogels depending on the sensitivity of drugs and the fabrication techniques [82]. Controlled release of loaded antibacterial materials is essential. It has been reported that drug release is limited by the skeletal structure of the aerogel; such that the release rate decreases with the prolongation of release time [83]. Wang et al. [84] reported that the change in the ratio of ingredients of the precursor materials would result in the aerogels with ordered pore structures and stable properties. In their research, the authors were able to control the pore size by changing the concentration of the polymer. Their result showed that higher concentration resulted in smaller pore size. Furthermore, the amount of drug released by aerogel was reported to be dependent on the pore sizes.

**Figure 5:**
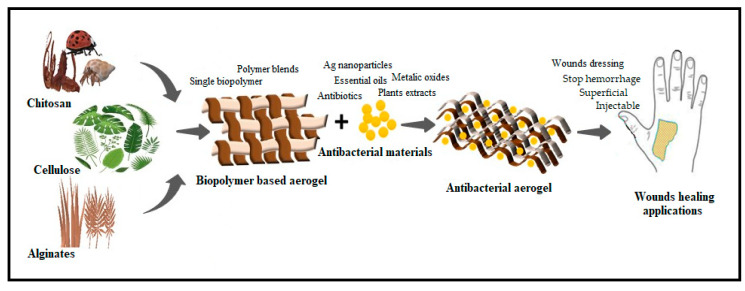
Schematic drawing of biopolymers antibacterial aerogels.

Another major factor that affects drug release is the specific surface area. The previous report showed that a change in the specific area resulted in a change in the dissolution rate of the drug and drug absorption in the body [85]. Biopolymer-based aerogels have an extremely large surface area. Thus, the adsorbed drug in aerogels exhibited low dissolution properties, and is useful for the long time delivery of drugs [86]. The surface chemistry of biopolymer-based aerogels also had an impact on the rate of drug release. Hydrophilic aerogels possess rapid drug release rate and serve as an advantage for the controlled release of poor water-soluble drugs. However, hydrophobic aerogels, in contrast, have a slower release rate, which is influenced by diffusion processes because they are more stable in water [87]. The hydrophilicity of natural polymers can be altered using chemical compounds [88]. The use of biopolymers as a carrier for different antibacterial substances including antibiotics [57], essential oils [89], plant extracts [90], enzymes [91], metallic oxides [92] and nanoparticles has been done [93]. Figure 5 presents a summary of the preparation process of antibacterial aerogel. 

Since the first aerogel was produced back in 1931, several other materials have been used for its production and applied in different applications. Table 2 presents a summary of the chronological development of antibacterial aerogels since its first invention. The utilization of aerogels in antibacterial delivery began recently at the beginning of this century. Silver was first used as an antibacterial agent in the aerogel [94]. The solution of silver nitrate (AgNO_3_) was used in the organic aerogels and reported to exhibit good antibacterial activity. Since then, various materials have been used as antibacterial activity as presented in Table 1.

### 3.1. Antibacterial Chitosan-Based Aerogels

**Figure 6:**
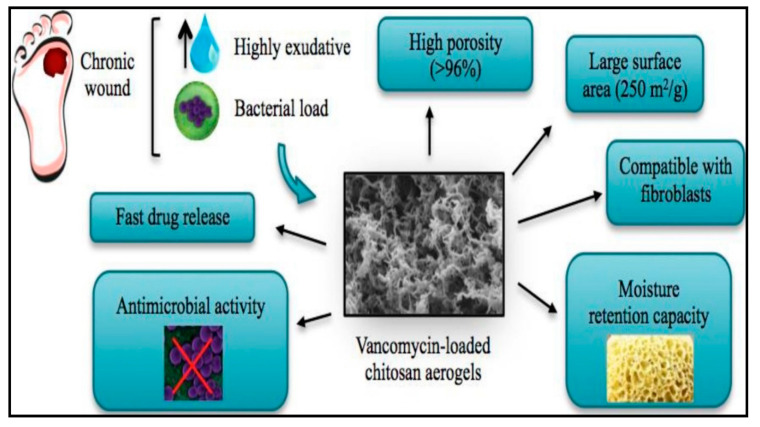
Properties of vancomycin-loaded chitosan aerogels, adapted from López-Iglesias et al. [105].

Chitosan has advantages over the other biopolymers in antibacterial activity due to the surface interaction between positively charged chitosan molecules and negatively charged membranes of microbial cells [107]. Raafat et al. [108] investigated the interaction between chitosan and bacterial membranes under a transmission electron microscope. The authors were able to identify chitosan molecules “as vacuole-like” attached on bacteria cell surfaces, and these vacuoles made the cell membrane of the bacterial cell became locally detached from the cell wall. The interaction between chitosan molecules and microbial membranes mediated by the electrostatic forces between the protonated NH^+3^ groups presents within the molecules of chitosan and the negative residues, presumably by competing with Ca^+2^ for electronegative sites on the membrane surface [109]. López-Iglesias et al. [105] evaluated the potential use of chitosan-based aerogel in delivering of vancomycin antibiotic for chronic wound management, and the loaded aerogel had unique properties (Figure 6). The authors reported that formation of aerogel enhanced air permeability and water sorption capacity of chitosan. The aerogel had high antibiotic content and fast drug release with enhanced antibacterial and cytocompatibility activities. Similarly, Wu et al. [110] loaded different antibiotics in chitosan aerogels. The ampicillin chitosan loaded aerogel exhibited strong antibacterial activity without any leaching. The authors also evaluated the in vitro cytotoxicity of their preparations using human cells, and all the aerogel composites proved to be biocompatible. Furthermore, the in vivo rate wound model analysis showed that the aerogel could speed up wound healing efficiently [110].

The synergistic antibacterial effect of the antibiotic and chitosan molecules give the aerogel stronger antibacterial activity against all tested microorganisms. Unlike the other antibiotic-loaded biopolymer-based aerogels, chitosan enhanced the impact of the antibiotics, giving strong antibacterial activity even after decreasing the dose. Chitosan aerogel microparticles have been used as a drug carrier for wound treatment applications and were fabricated through a new procedure [111]. Jet cutting technique with supercritical drying is used to manufacture chitosan-based aerogel with excellent textural properties and controlled release of antibacterial agents. In a different study, Gómez et al. [40] compared the antibacterial effect of chitosan-based aerogel when it was loaded with the antibiotic erythromycin and when it was loaded with elephant garlic extract. The antibacterial activity was measured prior to aerogel fabrication for both the antibiotic and elephant garlic extract. The plant extract resulted inactive against all tested bacteria, even methicillin-resistant *Staphylococcus aureus*, compared to erythromycin, which did not show any activity against this strain. The release of antibiotic from the aerogel to the agar was found to be dependent on the equilibrium forces with the biopolymer; in contrast, the diffusion of the active compounds of the plant extract was not dependent on the biopolymer content revealing a week interaction between chitosan and the plant extract. Yang et al. [112] used chitosan as a reinforcing agent with silver nanoparticles in micro and nano cellulose aerogels; both reinforcing agents dispersed well in the micro and nano cellulose aerogels, which expressed great antibacterial activity.

### 3.2. Antibacterial Cellulose Based Aerogels

Cellulose is the most used biopolymer carrier for different types of drugs; it has been used in antibacterial delivery to deliver different metallic oxides such as silver nanoparticles [113], copper, zinc oxide [114], and titanium dioxide [115]. The use of cellulose-based aerogel as the antibacterial carrier has been done by immobilizing or encapsulation of certain antibacterial material inside the cellulose network [89,116]. Various plant extracts and essential oils were reported to show strong antibacterial activity. *Punica granatum* peels extract has been reported to have stronger antibacterial activity than many common antibiotics [117]. Using such an extract together with cellulose-based aerogel can be of great interest as an antibacterial aerogel. Darpentigny et al. [116] used this principle. They extracted thymol, which is a natural compound from thyme that possesses strong antibacterial activity. The authors impregnated thymol with cellulose nanofibrils and produced aerogel with strong antibacterial activity against gram-positive and gram-negative bacteria. Similarly, Zhang et al. [89] used thyme essential oil in the cellulose-based aerogel, utilizing the advantage of having both antibacterial and antioxidant activity. The prepared aerogel exhibited good absorption capacity, good antibacterial activity, and sustained-release property. In another study, Khan et al. [91] used cellulose nanofibrils as aerogel matrix to immobilize silver nanoparticles and enzymes for antibacterial application. The authors tested their aerogel for potential use in clinical trials and it seemed to be non-toxic and biocompatible. Moreover, their report concluded that cellulose nanofibre is suitable to support for bioactive materials, effective in retaining enzymatic and antibacterial activities. The high cost of metallic nanoparticles limited their usage in research and prevented their commercialization. At present, most of the research is now focused on green alternatives such as plant extracts and/or essential oils.

Amoxicillin is one of the most used antibiotics for bacterial infection treatment. However, due to many reasons, including the use of minimal or less inhibitory concentrations, many bacteria have developed resistance to this antibiotic. Shan et al. [103] loaded amoxicillin in cellulose aerogel with the aim of getting the better performance of the antibiotic when it is directly applied to the infected area. The loaded aerogels exhibited excellent antibacterial activity with prolonged release of the antibiotic. The ability of cellulose to cross-link with other materials and make many surface interactions with polar and even non-polar compounds enhanced its potential for different biomedical applications [118]. A novel hybrid aerogel was fabricated by Zuguang et al. [119]. The aerogel was obtained by modification of the crystalline region of cellulose using the oxidation of some functional groups. The modified cellulose is then cross-linked with silver nanoparticles as an antibacterial substance. The obtained aerogel showed a strong antibacterial effect against many bacterial species [119]. Silver nanoparticles have been used as an antibacterial substance in many types of research, such as Salomoni et al., Tang et al., and Vijaya et al. [120,121,122], but due to some drawbacks, such as the costs and related health risks of silver nanoparticles, especially with high concentrations, meant they did not opt for large-scale industries [123]. However, using the particles in combination with modified aerogels minimizes the required dose and immobilizes the particles to avoid their release in the bloodstream upon using the aerogel for wound dressing, and thus minimizes or prevents the potential toxicity. Another cellulose-based wet-stabilized aerogel has been fabricated by Henschen et al. [102], with the porous structure of their CNF aerogel retained after soaking in water. The surface of this aerogel was able to adhere to more than 99% of bacteria. The surface modifications that they made to the aerogel were the main reason for bacterial adherence, which was confirmed by microscopy analysis [102]. Indicating the possibility of creating such 3D material able to high efficiency adsorb the bacteria, the high porosity, and thus great surface area of aerogel in combination with their open structure give the material potential for bacterial removal. The use of cellulose aerogels in antibacterial delivery has many environmental advantages of being green, sustainable, biodegradable, abundant, and non-environmentally taxing [124]. As cellulose molecules contain the preponderance of hydroxyl functional groups, they tend to possesses a strong affinity to water [125]. Water affinity or hydrophilicity of cellulose-based aerogel can be easily controlled, as it is able to crosslink with variety of different polarities of materials [126]. Despite its inherent hydrophilicity, cellulose and cellulose-based materials have unparalleled advantages as a precursor for potential production of hydrophobic materials. This material has been widely used in self-cleaning, self-healing, oil and water separation, electromagnetic interference shielding, etc. [127]. On the medical side, many studies evaluated the biocompatibility and cytotoxicity of cellulose-based aerogels [128,129,130,131], and it was concluded that no cytotoxicity has been observed with cellulose-based aerogels, confirming that they can be safely incorporated with antibacterial substances as a promising material for dressing and healing of wounds.

### 3.3. Antibacterial Alginates Based Aerogels

Alginates can react with polyvalent metal cations such as sodium and calcium ions [132]. These reactions often result in a 3D network of various materials such as hydrogels, microspheres, microcapsules, and aerogels. Martins et al. [133] evaluated the potential use of alginate aerogel in biomedical applications. Their report showed evidence of the high potential of these materials because of its biocompatibility and non-cytotoxicity. Unlike chitosan, this biopolymer does not exhibit any antibacterial activity, but it can form many surface interactions with different materials; thus, in most of the causes, alginate is used as a part of hybrid materials. Trucillo et al. [134] entrapped the antibiotic ampicillin loaded liposomes in alginate aerogels for prolonged drug release. The authors were able to make the release of antibiotic from the aerogel double the normal time. The multi free hydroxyl and carboxyl groups on the surface of alginate permit its cross-linking with several materials, which provides an outstanding candidate for antibacterial applications. Ma et al. [135] evaluated the potential use of alginate-based aerogel for wound dressing, using in vitro and in vivo methods. Their composite aerogels were formed from sodium alginate-graphene oxide and polyvinyl alcohol, in addition to norfloxacin, as an antibacterial material. The authors reported that the aerogel displayed excellent bioavailability of drug with sustained release behavior that was able to inhibit the growth of all tested bacteria. The aerogel enhanced the wound healing and promote the cell proliferation. Another alginate-based aerogel was formed by Raman et al. [52]. The aerogel was designed to carry calcium, zinc, and silver cations, which possess antibacterial and effective anti-inflammatory activity. These cations were suspended all over the huge surface area of aerogel. This resulted in its good homogeneity and strong antibacterial activity. This result showed that proper homogenization of the solution containing all the needed materials or cations prior to aerogel fabrication is important in order to get a homogeneous aerogel. The carboxylate groups of the guluronate in alginate polymer cross-link with different cations, which are immobilized within the polymeric network. Alginate and pectin core-shell aerogel beads have been produced by De Cicco et al. [57] for prolonging drug activity on wounds. The broad-spectrum antibiotic Doxycycline was suspended and immobilized in these aerogels, which had surprisingly high drug encapsulation efficiency (of up to 87%). The authors reported that drug release was dependent on the concentration of alginate and on drug/pectin ratio. The ability of pectin to interact with doxycycline makes the concentration of this drug go into the core, which mainly contain the pectin not alginate, giving another advantage of using biopolymer blends in term of delivering different drugs. 

### 3.4. Other Antibacterial Biopolymer-Based Aerogels

Carrageenan has been incorporated with cellulose and silver nanoparticles to form an antibacterial aerogel for wound healing applications [136]. Abdelgawad et al. used cellulose nanocrystals to enhance the mechanical properties of carrageenan aerogels and silver nanoparticles as antibacterial material. The release profile of the nanoparticles suggested that the aerogel showed good controlled and sustainable drug release. The aerogel was able to eliminate the growth of gram-positive and gram-negative bacteria, i.e., *Staphylococcus aureus* and *Escherichia coli,* respectively. Similarly, Shen et al. [119] successfully utilized bagasse to fabricate ultra-light aerogel, with silver nanoparticles as an antibacterial agent. The obtained aerogel exhibited a strong antibacterial effect, especially against *E. coli* and *Pseudomonas aeruginosa*. In a different study, pectin-based nanocomposite aerogels were fabricated for potential food packaging applications [62]. The authors’ incorporated titanium dioxide in the aerogel, and it displayed strong antibacterial activity against *E. coli.* Nisar et al. [137] used clove bud essential oil as antibacterial and antioxidant material in citrus pectin films. The inclusion of the essential oil significantly enhanced the water barrier properties of the films. However, the film showed complete growth of three common foodborne bacteria.

## 4. The Role of Biopolymer-Based Aerogel in Wound Healing Applications

Bacterial infections have been a major public health challenge associated with wounds and the leading cause of death. It has been reported that 1 out of 2% of the population in developed countries have suffered from a chronic wound in their lifetime, and this is even more for less developed countries [138,139]. In 2018, Sen [140] studied the estimated cost of human wounds and their burden. Sen reported that medicare cost projections for all wounds types ranged from $28.1 billion to $96.8 billion, including costs for infection management. The author also added that there was significant demand for wound care products. Globally, the annual average cost for wound care was $2.8 billion in 2014, and it will jump to $3.5 billion in 2021 [140]. In the design of wound dressings, preventing the infection is as important as stopping hemorrhaging and absorbing the wound exudates. Potential material must enhance cell proliferation, be easy to handle, biodegradable, permit gas exchange and be non-cytotoxic [141]. Being deprived of adequate oxygen, known as hypoxia, is one of the main factors hindering the process of wound healing, resulting in chronic wounds [142]. Contrasting many wound dressing materials, aerogel, with its high porosity, allows oxygen permeability, which is necessary for the normal healing process [143]. Hydrogel has been used in wound treatment as an antibacterial carrier and has been prepared from different polymeric materials [144]. Table 3 presents comparisons between hydrogels and aerogels in terms of antibacterial and wound healing applications.

**Figure 7:**
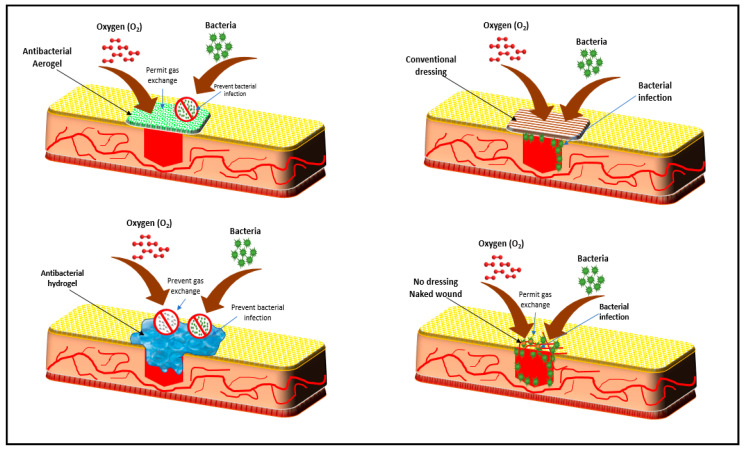
Schematic drawing of conventional dressing materials and antibacterial aerogel.

Over 300 wound dressing forms have been developed, but most of them have common problems associated with their poor absorption of wound exudates, poor gas exchange, lack of antibacterial activity, difficulty in removing and handling, and non-sterile and allergic reactions [141]. Traditional wound dressing materials are designed to keep the wounds dry and warm, such as absorbent pad, gauzes and felt [153]. One of the biggest issues associated with traditional dressings is the adherence structure, which sometimes causes real trauma to patients due to their difficulty to remove from the wound surface. However, some traditional dressings possess low adherence, such as those with polyamide contact layer [154]. Bacterial infection can possibly occur if no disinfection is used or the wound was not clean from microorganisms. Extensible bandages, cotton and rubber elastic bandages, elastic web bandages, elastic adhesive bandages, and rubber elastic are few examples of traditional wound dressing materials [155]. The unique properties of biopolymer-based antibacterial aerogels make them potential materials that can overcome all the problems associated with currently used wound dressing. Biopolymer-based aerogel have excellent absorption ability of wound exudates, strong antibacterial activity, and reduce adverse allergic effects. Furthermore, they are biocompatible and non-immunogenic; these properties improve the wounds healing process. Figure 7 presents comparative schematic drawings of using conventional wounds dressing and antibacterial aerogels. 

**Figure 8:**
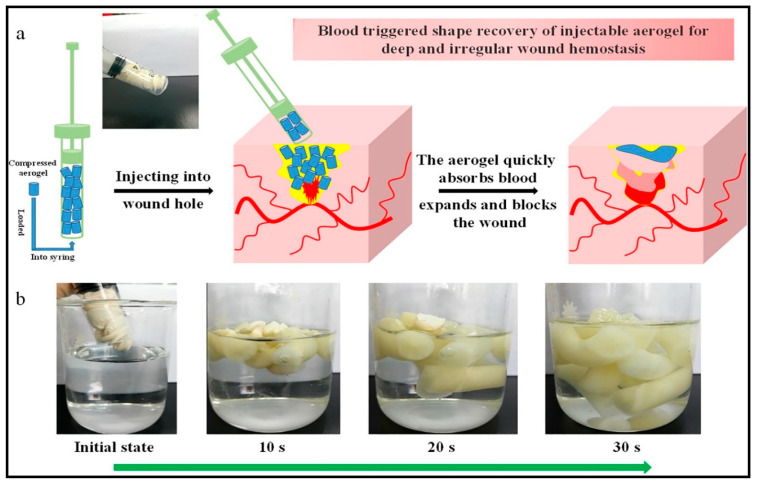
(**a**) Schematic illustration of simulating injection aerogel composites into the wound, (**b**) digital photos of aerogel with rapid shape recovery after water absorption. Adapted from Fan et al. [157].

Various antibacterial aerogels composites have been used in wound healing applications [156]. The ability of these aerogels to absorb water has been utilized in wound healing as small injectable pieces of aerogel, which can stop the bleeding of the deep wound and prevent bacterial infection at the same time. Recently, Fan et al. [157] fabricated an injectable aerogel composite for this purpose. The aerogel was composed of oxidized cellulose nanofiber to provide the physical and mechanical properties and chitosan for rapid hemostasis of non-compressible hemorrhage application, as Figure 8a presents. Unlike traditional wound dressing materials, using biodegradable and bioresorbable-based materials means that no further surgery is required to remove the products, because these materials are biodegradable, and some studies reported that they slowly degrade and are ultimately absorbed by the tissues in the body without any side effects [158,159]. Chitosan enhances the production of type III collagen production from the human cells around the wound environment, in addition to improving the proliferation and migration of fibroblasts. Thus it is considered the right choice in wound healing materials as it has all the properties that promote the healing grade [160].

In a different study, Batista et al. [132] developed a new preparation route for hybrid alginate-chitosan aerogel for wound healing applications. The authors observed no cytotoxic effect for the aerogel produced. The aerogel enhances wound recovery scratch compared with the control. It also possesses clear antibacterial activity against gram-positive and gram-negative bacteria. Surface modification of biopolymers has been reported to enhance its water absorption ability [147]. To enhance the absorption, Fan et al. used carboxyl groups to modify cellulose nanofiber with high-pressure homogenization and then reacted with the amidogen of chitosan to fabricate the injectable aerogels composites. These aerogels were observed to absorb the blood and rapidly expand to fill the wound area (Figure 8a,b) [157]. The aerogel had rapid shape recovery and good antibacterial ability due to the interaction between the two polymers and chitosan. However, this antibacterial activity could be significantly enhanced by the addition of an extra natural antibacterial material such as plant extract or essential oil. Cellulose/chitosan aerogel composite has been reported to be biocompatible and non-toxic to cells, with good adhesion and aggregation effect to red blood cells and platelets [2,149]. This is the same as many essential oils, which have been reported to have wound healing ability, antibacterial and antioxidant activities [161]. Alginate-chitosan aerogel is another composite aerogel that has been recently developed by Batista et al. [132]. The composite aerogel mix can provide a moisty environment for the alginate with the antibacterial activity of chitosan. These two factors are essential for the wound healing process. The composite aerogels were evaluated for potential use in the wound healing procedure. These composites were reported to be non-cytotoxic, biocompatible, and enhanced the closing of the wound area higher than the untreated control. The result showed that the combination of a biopolymer has great potential for wound healing applications [132]. In another study, Franco et al. [60] impregnated the mucopolysaccharide complex (Mesoglycan) onto calcium alginate aerogel. This is to produce novel material for re-epithelialization in the wound healing process. In vitro wound healing analysis confirmed that the aerogel composite increases the migration rate on fibroblast cells and enhances the proliferation of cells, which was observed from the result of covered distance for keratinocytes [60]. Biranje et al. [162] used chitosan/seaweed aerogel composite for wound dressing to promote hemostasis of the wounds and tissue growth, which is required during hemorrhaging. In vitro evaluation of hemostatic properties was done using human thrombin-anti-thrombin assay. The aerogel showed steady blood coagulation and adhesion of platelet and red blood cells, which helped in the formation of thrombin. The authors concluded that their dressing aerogel accelerated hemostatic activity against hemorrhaging. Chitosan-based aerogels and films have been fabricated for immediate hemorrhage control [163]. The aerogel exhibited mechanical strength, excellent fluid absorption, and improved blood clotting capacity. The authors performed intracellular reactive oxygen species assay and concluded that bacterial death resulted from damaging the cells. Similarly, chitosan/cellulose composite aerogel has been evaluated by Fan et al. [164] for massive hemorrhage control, which exhibited high water absorption capacity, excellent antibacterial activity, and rapid shape recoverability. The authors added that the developed aerogel had better coagulation ability than many traditional gauzes and gelatin sponge. Furthermore, the in vivo study showed rapid hemostasis, better biocompatibility, and no cytotoxicity was observed [164]. In summary, three-dimensional biopolymer-based aerogels have been reported as an ideal candidate to replace traditional wound dressings. Some previous studies in this area showed that biopolymer-based aerogel has an excellent potential for antibacterial delivery for superficial wound dressing and injectable for to stop hemorrhage.

The wound healing process is a multi-factorial physiological process; the complexity of its different phases and undesired abnormalities could make the healing process very difficult. The wound dressing is designed to prevent bacterial infection because this is one of the most important external effectors during the healing process of wounds. There is a growing interest in developing smart wound dressings characterizes with: self-sterilization and inhibition of bacterial invasion, bio-adhesiveness to the wound surface, ease of application, biodegradability, oxygen permeability, and non-toxicity, etc. The current biopolymer-based antibacterial aerogels have the potential to be the first option in wound dressing to prevent the chronicity of wounds and enhance the quality of patients’ lives. The in vivo results of many studies in term of antibacterial wound healing and stopping hemorrhaging are promising for many wound healing applications.

## 5. Challenges and Propositions of Biopolymer-Based Antibacterial Aerogels

The multi-functional groups surface modification of biopolymers and the high surface area of nanomaterial aerogels give the nano biopolymer-based aerogels tremendous ability to form cross-links with different functional groups. Little research has been done regarding the incorporation of plant extracts and/or essential oils in aerogels. However, the investigations of the antibacterial activity of many essential oils, plants aquatic and alcoholic extracts has been done, and many of them proved their strong antibacterial activity. In alternative medicine, plant extracts and essential oils have been reported to have hemostasis ability as well as antimicrobial and antioxidant activity [165]. Antibacterial cellulose-based edible films have been fabricated for food packaging applications by using thyme essential oil as an antibacterial ingredient inside the film [166]. The essential oil forms many cross-links all along the surface of cellulose film, giving the film the antibacterial character and keeping the physicochemical and mechanical properties of cellulose film. The same strategy could be followed in biopolymer-based aerogels, and the huge surface area of this material, numerous properties, such as antibacterial, antioxidant, and/or anti-inflammatory substances could be integrated inside these aerogels. The same principle would be helpful in developing chronic diabetic wound sheets; the air permeability of aerogel will provide extensive oxygen on the surface of the wound, preventing the growth of anaerobic bacteria, which is the main cause of gangrene [167]. Antibacterial aerogels will provide clean wounds and inhibit the aerobic bacterial growing, without closing or preventing the aeration, which is necessary for wound healing [168]. The large-scale production and commercialization of biopolymer-based antibacterial aerogels have not yet started. It is still in pre-clinical and clinical evaluation. As wound dressing is of high demand, the cost of production of biopolymer-based antibacterial aerogel will be reduced compared to the conventional material because all the logistics, environmental issues and post wound healing costs would have been eliminated. Furthermore, that large-scale production of biopolymer-based antibacterial aerogels will significantly lower the cost of production.

However, in order to propose the safe use of biopolymer-based aerogels for any medical application, it is important to evaluate short-term as well as long-term toxicity and fate of these materials. Recently, some authors raised important concerns that justify the study of bio-interactions and the possible impact on humans upon the exposure to nanocellulose materials [169,170,171]. This would provide consistent and useful knowledge that can guide the outgrowth of regulations. Most of past and current health impact evaluations have been studied by the determination of the viability and cytotoxicity of the material on animal or human cell lines. However, this is done without considering the chronic effect of the material during the long-term exposure or exacerbation of pre-existing disease conditions. Furthermore, such studies have not been conducted on the cytotoxicity of reported potential blends of biopolymer-based aerogel. Different fabrication techniques, in most cases, have been followed using different chemicals and procedures, raising another concern about the effect of each technique on the antibacterial functionality of the developed material. The intensity of concerns about the developing of antibiotic resistance has depended on various factors, including bacterial exposure to different materials. Yahya et al. [172] evaluated the effect of some natural materials on the antibiotic sensitivity of bacteria and concluded a significant increase upon the exposure. Pan et al. [173] reported that some metal nanoparticles induced mutagenic effect on bacterial cells. The effect of microbial exposure to nano polymer-based materials is still under research and not yet been determined. Further studies are needed on the effect of biopolymer-based aerogels on the microorganisms, to determine the potential mutagenic induction.

## 6. Conclusions

This review has critically analyzed the properties of three biopolymer-based aerogels used in antibacterial delivery and highlighted the most recent works exploring the utilization of these aerogels in wound healing and skin tissue repair applications. Biopolymer-based aerogels based on previous studies have provided a potential platform to cross-link with a large variety of antibacterial materials to exploit new forms of functional materials. The quality of wound treatment process, especially chronic wounds, have been proposed for improvement using biopolymer-based aerogel. Biopolymer-based aerogels are suitable for these biomedical applications due to their ability to be incorporated with different antibacterial materials, resulting in aerogels with antibacterial properties. The utilization of antibacterial biopolymer-based aerogels in wound healing applications has been steadily raised due to the increasing demand for safer medical solutions in term of chronic wound healing, stopping hemorrhaging and enhance cell proliferation and tissue repair. Many studies have confirmed that biopolymer-based aerogels are biocompatible, non-cytotoxic and do not cause any immune reaction. However, much work is still needed in the application of the aerogel to different types of wound. Furthermore, different body allergies need to be considered before its commercialization.

## Figures and Tables

**Table 1:** Biological, wound healing properties and molecular structures of chitosan, cellulose and alginate.

Biopolymer	Biological Properties	Molecular Structure	References
Chitosan	- Hemostatic agent due to positive charges that can bind to negative charges on red blood cells. - Antibacterial and anti-fungal. - Mucoadhesive properties - Wound healing acceleration and immune system stimulation.	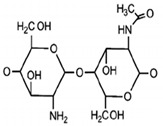	[29]
Cellulose	- High water absorption and holding capacities. - Good wound exudates drainage capacity. - Support and enhance the growth and proliferation of cells.	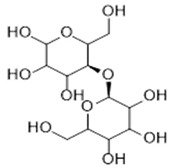	[30]
Alginates	- Preserving a solid-like attribute at acidic conditions. - Hemostatic properties, which are useful for bleeding wounds. - Good mucoadhesive properties. - Barrier protects immobilized material toward physical stress.	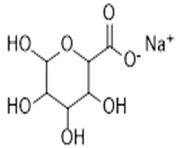	[31]

**Table 2:** Chronological development of the antibacterial aerogels.

Year	Type of Aerogels	Remark/Incorporated Antibacterial Material	Reference
1931	Silica aerogel	The first invention of aerogel.	[14]
1968	Metal oxide and silica	Development of sol-gel route for aerogel fabrication.	[95]
1989	Polymer aerogels	Organic and carbon aerogel using sol-gel route.	[96]
1997	Polymer aerogels	Ultralight aerogels using cross-linking techniques.	[97]
2006	Carbon-based aerogels	Silver as antibacterial material and direct immersion of organic aerogels in aqueous AgNO3 solutions.	[94]
2008	Nanocellulose-based aerogel	The first use of silver nanoparticles for antibacterial properties in nanocellulose aerogel.	[93]
2011	Chitosan aerogels	Antibacterial mesoporous pure chitosan aerogels.	[37]
2013	Nanofibrillated cellulose aerogel	Iron oxide and silver nanoparticles dispersed in nanofibrillated cellulose aerogel.	[98]
2013	Silica alcogel	Essential oils of medicinal plants used as antibacterial materials for air and surface disinfection.	[90]
2014	Cellulose-based aerogel	Lysozyme, Zinc oxide and gold nanoparticles immobilized in the cellulose network as antibacterial agents.	[92,99]
2014	Cellulose diacetate fibers	Bacteriophage used as an antibacterial agent, which encapsulate within the cellulose fibers.	[100]
2015	Cellulosic-based paper	Triclosan used as antibacterial material, in addition to improving the strength.	[101]
2016	Cellulose aerogels	Layer-by-layer surface-modified cellulose aerogel was able to adhere to bacterial cells from aquatic solutions.	[102]
2017	Nanocellulose aerogels	Lysozyme enzyme immobilized inside the cellulose aerogel as antibacterial material.	[91]
2018	Cellulose-based aerogels	Amoxicillin and gentamicin antibiotics loaded in the microcrystalline cellulose network.	[103,104]
2019	Chitosan aerogel	Vancomycin antibiotic-loaded inside the chitosan aerogel.	[105]
2020	Bacterial cellulose aerogel	Modified cellulose with N-isopropyl acrylamide, which used as an antibacterial agent after enduring chlorination.	[106]
2020	Nanocellulose aerogel	Essential oils as a natural antibacterial agent in the aerogel.	[89]

**Table 3:** Comparison between hydrogel and aerogel in wound healing applications.

Functionality	Hydrogels	Aerogels	Reference
Fluids absorption in wet wounds	Does not absorb fluids and cannot be used in wet wounds.	Able to absorb large amounts of fluids in wet wounds.	[145,146]
Stop hemorrhage	Not applicable.	Can be used to absorb fluid and expand to fill the wound.	[147]
Mechanical stability	Poor mechanical strength and liable to tearing easily.	Excellent mechanical strength and easy to handle.	[148]
Wounds aeration	Does not provide aeration to wounds.	The high porosity of aerogels provides suitable aeration for wounds.	[149]
Potential cytotoxicity	Relatively higher	Lower cytotoxicity.	[150]
Reliability for patients	Difficult for patients to change their dressings.	Easy for patients to change their dressings.	[151]
Cost of production	Lower cost of production.	Higher cost of production.	[152]
